# Lymph-Targeted Delivery of CUR-NLCs Enhances Oral Bioavailability: Evidence from a Double-Catheterized Rat Model

**DOI:** 10.3390/pharmaceutics17111484

**Published:** 2025-11-17

**Authors:** Haoming Chi, Xiaorui Zhang, Zhiyuan Chen, Qiuyong Chen, Bo Yang, Hui Deng, Daojin Yu

**Affiliations:** 1 Fujian Key Laboratory of Traditional Chinese Veterinary Medicine and Animal Health, College of Animal Sciences, Fujian Agriculture and Forestry University, Fuzhou 350002, China; 15739856691@163.com (H.C.); xiaorui.zhang@sibcb.ac.cn (X.Z.); czhiyuan20230228@yeah.net (Z.C.); fjchenqiuyong@163.com (Q.C.); ybvet@fafu.edu.cn (B.Y.); deng12345hui@163.com (H.D.); 2 Fujian Academy of Agricultural Sciences Institute of Animal Husbandry and Veterinary Medicine, Fuzhou 350002, China; 3 Animal Core Facility, CAS Center for Excellence in Molecular Cell Science, Shanghai Institute of Biochemistry and Cell Biology, Chinese Academy of Sciences, University of Chinese Academy of Sciences, Shanghai 200031, China

**Keywords:** curcumin, nanostructured lipid carriers (NLCs), oral bioavailability, lymphatic transport, pharmacokinetics

## Abstract

**Background/Objectives:** Curcumin (CUR), a natural polyphenol with poor solubility and significant first-pass metabolism, shows extremely low oral bioavailability. Although CUR-loaded nanostructured lipid carriers (CUR-NLCs) have demonstrated potential in enhancing oral absorption, direct evidence regarding their intestinal lymphatic transport mechanism remains insufficient, and current understanding largely relies on indirect speculation. **Methods:** CUR-NLCs were prepared by emulsion-ultrasonication and evaluated for their physicochemical properties including particle size, zeta potential, polydispersity index, encapsulation efficiency, drug loading, stability and release profile. A mesenteric lymph duct-jugular vein shunt rat model combined with transmission electron microscopy was employed to assess the pharmacokinetic behavior and lymphatic transport pathway. **Results:** CUR-NLCs had a mean size of 117.28 ± 1.32 nm, 99.99% encapsulation efficiency, and 1.73% drug loading. They exhibited good gastrointestinal stability and sustained release (<55% in 24 h). CUR-NLCs significantly enhanced oral absorption versus free CUR, with 5.13-fold higher relative bioavailability, 5.25-fold greater C_max_, and extended half-life (33.49 ± 3.15 h). CUR was detected only in the lymph of the CUR-NLCs group, confirming intestinal lymphatic transport. TEM revealed abundant chylomicrons (0.1–2 μm) in jejunal epithelial cells, providing morphological support. **Conclusions:** This study directly demonstrates that CUR-NLCs improve oral bioavailability via intestinal lymphatic absorption, offering a viable strategy for delivering poorly soluble hydrophobic drugs.

## 1. Introduction

Curcumin (CUR) is a natural hydrophobic polyphenol compound extracted from the rhizomes of *Curcuma longa* L. [1]. Due to its antioxidant [2], anti-inflammatory [3], antitumor [1], and low-toxicity properties [4], it is widely used in medicine, supplements, and the dietary industry [5]. However, as a Class IV drug under the Biopharmaceutics Classification System, CUR exhibits low solubility, low permeability, and poor stability in the gastrointestinal tract [6]. Moreover, it undergoes rapid metabolism and exhibits a short half-life in vivo [7], collectively resulting in exceptionally low oral bioavailability and thus limiting its clinical utility. For example, when humans take 12 g of CUR orally, the maximum blood concentration is only 0.05 μg/mL [8]. Therefore, finding effective strategies to improve the oral bioavailability and safety of CUR has become a major research focus.

To overcome these limitations, researchers have explored various formulation strategies, among which lipid-based nano-delivery systems have demonstrated unique advantages. These systems undergo enzymatic digestion in the gastrointestinal tract to form mixed micelles, significantly enhancing the solubility, permeability, and absorption of poorly soluble drugs [9]. Several lipid-based delivery systems have been developed to date, including nanoemulsions [10], self-emulsifying drug delivery systems [11], and nanostructured lipid carriers (NLCs) [12]. Among them, NLCs, as second-generation lipid nanocarriers, possess a unique “solid lipid matrix + liquid oil phase” architecture that gives rise to an imperfect lattice structure. This structural feature confers several advantages, such as high drug loading capacity, high encapsulation efficiency, robust physical stability, and good biocompatibility [13,14,15], thus establishing NLCs as an ideal platform for improving the oral delivery of CUR. Consistent with these attributes, CUR-NLCs have been shown to markedly improve plasma levels and bioavailability. For example, taurocholate-modified CUR-NLCs enhanced the relative bioavailability of CUR by 4.27-fold compared to the free drug [16], while Dolatabadi et al. reported a 3.41-fold increase with their CUR-NLC formulation [17].

Although the pharmacokinetic benefits of CUR have been established, the underlying absorption mechanism, particularly the role of intestinal lymphatic transport in the uptake of CUR-NLCs, remains poorly understood and is primarily inferred from indirect evidence. Current research largely depends on indirect methods such as plasma pharmacokinetic profiles or static tissue analysis to speculate on lymphatic transit. For example, Baek et al. [18] measured CUR levels in mesenteric lymph node homogenates to suggest lymphatic uptake. While this static approach confirms nodal accumulation, it cannot track dynamic drug levels in lymph or distinguish between systemic lymphatic absorption and mere nodal retention. In another study, Wang et al. [19] used cycloheximide (CHM) to suppress lymphatic function and observed approximately 50% decrease in the relative bioavailability of CUR-loaded micelles, attributing this to blocked lymph transport. However, CHM may non-specifically disrupt enterocyte metabolism, confounding the interpretation of bioavailability changes. Moreover, without direct measurement of drug concentration in lymph, the actual contribution of the lymphatic route remains unquantified. This reliance on indirect inference, but not direct quantification, significantly hinders a mechanistic understanding of CUR-NLCs absorption and impedes rational formulation development and clinical translation.

## 2. Materials and Methods

### 2.1. Materials

#### 2.1.1. Chemical and Major Instruments

CUR, pepsin, pancreatin, Tween 80, and analytical grade chemicals were obtained from Titan Tech and Sinopharm (Beijing, China); H125 Solid–liquid Lipid Mixture provided by Yu-Lab, Fujian Agriculture and Forestry University; chromatographic acetonitrile and Zoletil 50 were purchased from Sigma-Aldrich (St. Louis, MO, USA) and Virbac (Carros, France). Key instruments such as transmission electron microscope (FEI, Hillsboro, OR, USA), LC-MS/MS (Sciex, Framingham, MA, USA), centrifuges (Eppendorf, Germany), and ultrasonic cell disruptor (Xinzhi, Shumei, Beijing, China) were used. Rat surgical devices including vascular access buttons, catheters, and sampling accessories were supplied by Instech (Plymouth Meeting, PA, USA).

#### 2.1.2. Liquid Chromatography-Tandem Mass Spectrometry (LC-MS/MS) Conditions

An ACQUITY UPLC BEH C18 VanGuard precolumn (2.1 mm × 5 mm, 1.7 µm) was used with a flow rate of 0.3 mL/min, column temperature of 40 °C, and injection volume of 2 μL. The mobile phase consisted of acetonitrile with 0.1% formic acid and aqueous 0.1% formic acid in a ratio of 50:50 (*v*/*v*). Detection was performed in multiple reaction monitoring (MRM) mode using a transition of *m*/*z* 367.1→217.2 for quantification.

#### 2.1.3. Animals

This study used 24 Specific Pathogen Free (SPF) grade male Sprague Dawley (SD) rats (9 weeks old, weighing 280 ± 9 g) and 18 SPF-grade male ICR mice (8 weeks old, weighing 22 ± 3 g). All animals were purchased from Zhejiang Vital River Laboratory Animal Technology Co., Ltd. (Jiaxing, China) [SCXK (Zhe) 2019-0001], with a quality certification number of 20211221Aazz0619000169. The rats and mice were housed in the experimental animal barrier facility of the Center for Excellence in Molecular Cell Science, Chinese Academy of Sciences [SYXK (Shanghai) 2018-0007], with free access to water and food. The environmental conditions were maintained at 20–25 °C, 40–70% relative humidity, noise ≤ 60 dB, and lighting at 20 lx (12/12 h light-dark cycle). The animals were housed 3 rats per cage and 5 mice per cage, with a 7-day acclimatization period before the experiments began. The animal experimental protocol was reviewed and approved by the Institutional Animal Care and Use Committee (IACUC) of the Center for Excellence in Molecular Cell Science, Chinese Academy of Sciences (IACUC No. SIBCB-S118340-2112-045, approved on 12 January 2024).

### 2.2. Preparation of CUR-NLCs

CUR-NLCs were prepared using the emulsion-ultrasonication method [20]: CUR (5 mg), H125 solid–liquid lipid mixture (glyceryl dibehenate, glyceryl monooleate, phosphatidylcholine, 400 mg), Tween 80 (200 mg) were dissolved in methanol. The solvent was removed by rotary evaporation at 75 °C in a water bath to form a lipid film. Deionized water was added, and the mixture underwent pulsed sonication in an ultrasonic cell disruptor, yielding a final CUR-NLCs concentration of 0.5 mg/mL. Blank NLCs were prepared identically except without CUR addition.

### 2.3. Physicochemical Characterization of CUR-NLCs

#### 2.3.1. Particle Size and Zeta Potential Measurement

The average particle size, polydispersity index (PDI), and zeta potential of Blank-NLCs and CUR-NLCs (CUR concentration: 0.1 mg/mL) were determined using dynamic light scattering (DLS) [21] (Mastersizer Ultra laser particle size analyzer, Malvern Panalytical, Malvern, UK). Each sample was measured in triplicate.

#### 2.3.2. Encapsulation Efficiency (EE) and Drug Loading (DL)

Free drug content was separated via ultrafiltration centrifugation [20] (100 kDa, 3000 rpm, 4 °C, 15 min), and CUR concentration was quantified by LC-MS/MS. Drug loading and encapsulation efficiency were calculated using standard formulas. The calculation formulas for DL and EE are:

$$\begin{array}{c} DL(\%)=\frac{W_{total\;drug}-W_{free\;drug}}{W_{total\;lipid}+W_{drug\;encapsulaed\;in\;formulation}}\times 100\\ EE(\%)=\frac{W_{total\;dnug}-W_{free\;drug}}{W_{total\;drug}}\times 100 \end{array}$$

Among these, W_total drug_ represents the amount of CUR in the formulation, W_free drug_ represents the amount of free CUR in the formulation, W_total lipids_ represents the amount of lipids used to prepare the formulation, and W_drug encapsulated in formulation_ represents the amount of CUR encapsulated within the formulation.

#### 2.3.3. Transmission Electron Microscope (TEM) Morphological Observation

Blank-NLCs and CUR-NLCs (0.01 mg/mL) were deposited onto copper grids, negatively stained with 2% uranyl acetate, and observed under TEM (Hitachi 3H-7000FA, 75 kV, Hitachi, Ltd, Tokyo, Japan).

### 2.4. Detection of CUR-NLCs Stability

#### 2.4.1. Storage Stability

CUR-NLCs were stored at 25 °C (room temperature) and 4 °C (refrigeration) for 15 days, protected from light. Particle size, PDI, and zeta potential were measured on days 0, 7, and 15 using DLS, with triplicate measurements for each condition.

#### 2.4.2. CUR-NLCs Gastrointestinal Stability

The gastrointestinal stability of CUR-NLCs was evaluated by incubating the nanoparticles in simulated gastric fluid (SGF, pH 1.2) and simulated intestinal fluid (SIF, pH 6.8) at 37 °C under continuous agitation (100 rpm) [22]. Samples were collected at predetermined time intervals (0, 0.5, 1, 2, 4 h for SGF; 0, 0.5, 1, 2, 4 h for SIF) and analyzed for particle size, PDI, zeta potential, and drug retention rate using DLS and LC-MS/MS.

#### 2.4.3. CUR-NLCs Release

The release behavior of CUR-Sol and CUR-NLCs was evaluated using a dynamic dialysis method [23]. Samples equivalent to 100 μg of CUR from either CUR-Sol or CUR-NLCs were placed in pre-treated cellulose ester dialysis bags (molecular weight cutoff: 14 kDa). The bags were immersed in 20 mL of phosphate buffer (10 mM, pH 6.8) containing 0.5% (*w*/*v*) Tween 80 and incubated in a constant-temperature water bath shaker (37 °C, 100 rpm). At predetermined time points (0.5, 1, 2, 4, 8, 12, and 24 h), the entire release medium was withdrawn and immediately replaced with an equal volume of fresh medium. The collected release samples were centrifuged (12,000 rpm, 10 min), and the supernatant was analyzed by LC-MS/MS to determine the CUR content. The cumulative release of the drug was calculated accordingly.

$$\mathrm{Cumulative}\;\mathrm{Release}\;\mathrm{Rate}(\%)=\left(\frac{\sum_{i=1}^{n}C_{i}\times V}{M_{0}}\right)\times 100\%$$

Among these, C_i_ represents the concentration of CUR in the release medium at the i-th sampling time point, V represents the volume of the release medium, and M_0_ represents the initial total amount of CUR loaded in the dialysis bag.

### 2.5. Lymphatic Transport and Pharmacokinetic Analysis of CUR-NLCs

#### 2.5.1. Establishment of an Auxiliary Mesenteric Lymph-Jugular Venous Reflux Model in SD Rats

Twenty-four SPF male SD rats were intramuscularly injected with Zoletil 50 (50 mg/kg) for anesthesia. After confirming the onset of anesthesia (decreased respiratory rate, good muscle relaxation, and loss of pain reflex), they were secured on a surgical table. The abdominal area was shaved and disinfected with 75% ethanol.

Jugular vein catheterization: An incision was made in the left clavicular region of the neck. The external jugular vein was isolated, and its wall was incised at a 45° angle. A venous catheter was inserted (advanced approximately 3.5 cm into the left subclavian vein), secured, and connected to the jugular vein port of the Vascular Access Button (VAB). The VAB was then docked with the commutator, and the incision was sutured layer by layer.

Mesenteric lymph duct catheterization: A midline incision was made along the upper two-thirds of the abdominal midline. The peritoneal cavity was opened to expose the area of the left renal vein and inferior vena cava. The mesenteric lymph duct, running parallel to the mesenteric artery, was identified. Under a stereomicroscope, the mesenteric lymph duct was carefully isolated. Its wall was incised at a 45° angle, and a lymph catheter was inserted (advanced approximately 1 cm). The catheter was ligated and secured to the distal end of the lymph duct. The other end of the catheter was tunneled subcutaneously to the neck and connected to the lymphatic port of the VAB.

Awake and mobile device connection: The VAB, a 2-channel VAB tether, rotor, multi-axis balance arm, and sampling cage were connected. The collection catheter was positioned at the height of the lymphatic catheter.

Postoperative recovery: The animals were individually housed and allowed to recover for 7 days with free access to water. A soft diet was provided for the first 3 days post-operation. To ensure postoperative welfare, analgesia was provided by intramuscular injection of flunixin meglumine at a dose of 2 mg/kg every 12 h for 3 days. Additionally, antimicrobial prophylaxis was administered via subcutaneous injection of enrofloxacin at 2.5 mg/kg once daily for 3 days. To minimize suffering, humane endpoints were predefined. Any animal exhibiting signs such as >20% body weight loss, severe anorexia, profound weakness, major trauma, secondary infection, a moribund state, or severe, treatment-refractory organ dysfunction was humanely euthanized via CO_2_ asphyxiation. This decision was also made based on a poor prognosis assessment by the facility veterinarian [24].

#### 2.5.2. Pharmacokinetic Analysis in Serum and Lymph

Twelve rats successfully established with the single jugular vein cannulation model were randomly divided into 2 groups (*n* = 6 per group, CUR-Sol group and CUR-NLCs group). After fasting for 12 h, the animals were orally administered a dose of 5 mg/kg via gavage. While conscious, blood samples (50 μL) were collected from the jugular vein catheter at 0.033, 0.083, 0.25, 0.5, 1, 2, 4, 8, 12, and 24 h after administration and placed in heparin sodium anticoagulant tubes. The plasma was then separated by centrifugation at 4000 rpm and 4 °C. Twelve rats successfully established with the mesenteric lymph duct-jugular vein assisted return model were randomly divided into 2 groups (*n* = 6 per group, CUR-Sol group and CUR-NLCs group). After fasting for 12 h, the animals were orally administered a dose of 5 mg/kg via gavage. While conscious, lymph fluid was continuously collected during the time intervals of 0–4 h, 4–8 h, and 8–24 h after administration. To prevent fluid imbalance caused by the lymph collection procedure, an equivalent volume of Ringer’s solution was promptly supplemented through the jugular vein catheter. At the end of the experiment, all rats were subjected to euthanasia by carbon dioxide (CO_2_) asphyxiation. Due to the technical nature of the surgical procedures, the researchers performing the model establishment and drug administration were necessarily aware of the group allocations. However, to minimize bias, blinding was implemented during the outcome assessment and data analysis phases. Specifically, the laboratory analysts responsible for quantifying drug concentrations in plasma and lymph samples were blinded to the group identity (CUR-Sol vs. CUR-NLCs) of each sample. Furthermore, the statisticians who performed the pharmacokinetic analysis received coded data that concealed the group assignments until after the final statistical comparisons were completed.

#### 2.5.3. TEM Observation of Intestinal Segments

A total of 18 SPF male ICR mice were randomly divided into 3 groups (*n* = 6 per group, NA group, CUR-Sol group, and CUR-NLCs group). The CUR-Sol and CUR-NLCs groups were orally administered 10 mg/kg of CUR-Sol and CUR-NLCs, respectively, while the NA group received an equal volume of normal saline. The mice were euthanized by CO_2_ asphyxiation 5 min after administration, and jejunal tissues were collected, rinsed with pre-cooled normal saline, and cut into sections. The tissues were fixed with 2.5% glutaraldehyde at 37 °C for 1 h, followed by fixation at 4 °C for 12 h. Subsequent processing included rinsing, post-fixation, dehydration, embedding, and ultrathin sectioning. Finally, the samples were observed under an 80 kV TEM.

### 2.6. Statistical Analysis

The pharmacokinetic parameters of CUR, including maximum plasma concentration (C_max_), time to reach Cmax (T_max_), elimination half-life (T_1/2_), area under the concentration-time curve (AUC_0–t_), and relative bioavailability (AUC_0–∞_), were calculated from the mean plasma concentration-time data using WinNonlin 6.2 software (non-compartmental model, Certara, Radnor, Pennsylvania, USA). Concentration data below 80% of the lower limit of quantification were excluded from pharmacokinetic parameter calculations. Statistical analysis was performed using GraphPad Prism 8.0 software (GraphPad Software, San Diego, California, USA), with one-way analysis of variance (ANOVA) for multiple group comparisons and LSD-t test for pairwise comparisons to evaluate the bioequivalence of the formulations. All raw data were analyzed using one-way ANOVA (after confirming assumptions of normality by the Shapiro–Wilk test and homogeneity of variances by Bartlett’s test), followed by Tukey’s multiple-range tests. For data that did not meet these assumptions, non-parametric equivalents were used. Statistical significance was defined as * *p* < 0.05 and ** *p* < 0.01.

## 3. Results

### 3.1. Characterization of CUR-NLCs

**Figure 1:**
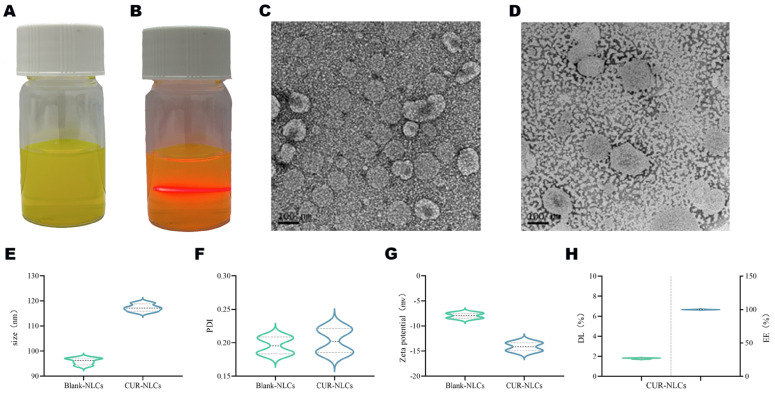
(**A**): CUR-NLCs; (**B**): Transmission image showing the path of infrared laser beam through the sample; (**C**): TEM image of Blank-NLCs; (**D**): TEM image of CUR-NLCs; (**E**): Particle size distribution of Blank-NLCs and CUR-NLCs; (**F**): PDI of Blank-NLCs and CUR-NLCs; Note: In Figures (**E**,**F**), the dashed lines with different widths correspond to the two formulations respectively: the dashed line with the narrower width represents the box plot interval of Blank-NLCs, while the dashed line with the wider width represents the box plot interval of CUR-NLCs; the area enclosed by the dashed lines corresponds to the interquartile range of each group’s data, which is used to distinguish the statistical distribution characteristics of the two experimental groups in terms of particle size (**E**) and polydispersity index (**F**); (**G**): Zeta potential of Blank-NLCs and CUR-NLCs; (**H**): Encapsulation efficiency(Right of the dotted line) and drug loading (Left of the dotted line)capacity of CUR-NLCs.

This study successfully prepared CUR-NLCs using an emulsion-ultrasonication method. The resulting formulation appeared as a homogeneous, translucent emulsion exhibiting characteristic yellow opalescence under natural light (Figure 1A). The Tyndall effect was observed when an infrared laser beam passed through the CUR-NLCs system, producing a well-defined red-light path without localized spot distortion (Figure 1B), confirming the successful preparation of monodispersed NLCs with a particle size below 200 nm. TEM observations were consistent with dynamic light scattering measurements, revealing that both Blank-NLCs (Figure 1C) and CUR-NLCs (Figure 1D) exhibited typical spherical morphology with particle sizes around 100 nm.

Particle size analysis indicated that both Blank-NLCs and CUR-NLCs had an average diameter of approximately 100 nm with PDI below 0.3, demonstrating good dispersity (Figure 1E,F; Table 1). Zeta potential measurements showed values of −7.93 ± 0.33 mV for Blank-NLCs and −14.14 ± 0.30 mV for CUR-NLCs, indicating an increase in absolute potential value (Figure 1G; Table 1). The drug loading capacity of CUR-NLCs reached 1.73%, and the encapsulation efficiency was nearly theoretical maximum at 99.99% (Figure 1H; Table 1).

### 3.2. Stability and In Vitro Release Behavior of CUR-NLCs

**Figure 2:**
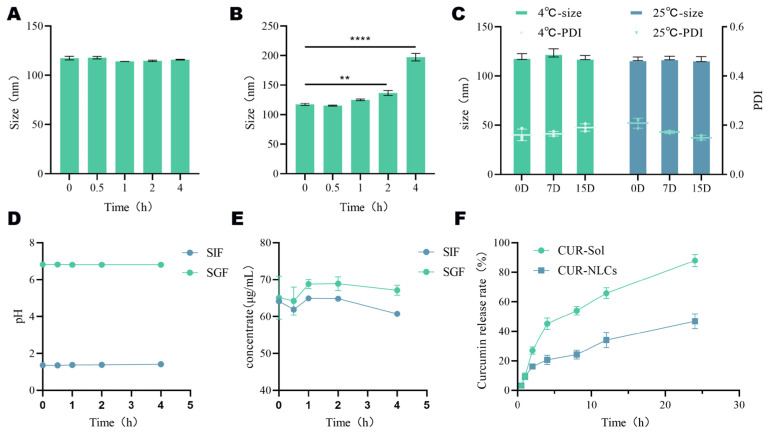
(**A**): Gastric stability of CUR-NLCs; (**B**): Intestinal stability of CUR-NLCs; (**C**): pH value of CUR-NLCs in gastric fluid and intestinal fluid; (**D**): Drug content of CUR-NLCs in gastric fluid and intestinal fluid; (**E**): Storage stability of CUR-NLCs; (**F**): In vitro release rate. Note: ** *p* < 0.01 and **** *p* < 0.0001.

In vitro gastrointestinal stability tests indicated that CUR-NLCs exhibited good physical stability in simulated gastric fluid. During the 4 h incubation, the average particle size remained within the range of 109.45 ± 1.77 nm to 117.46 ± 1.80 nm (Figure 2A), with fluctuations within 8 nm and no significant differences between groups (*p* > 0.05). The pH variation of the system was controlled within 0.05 units, also showing no significant intergroup differences (*p* > 0.05, Figure 2D). The concentration of CUR in gastric juice shows biphasic kinetic characteristics, featuring an initial decrease, a brief increase at 0.5–1 h, and then another decrease. Yet, drug content showed no significant change post- vs. pre-incubation (*p* > 0.05, Figure 2E). indicating that CUR-NLCs maintained structural integrity in the gastric environment.

However, the stability of CUR-NLCs changed significantly in simulated intestinal fluid. After 2 h of intestinal fluid incubation, the average particle size increased significantly from an initial 117.47 ± 1.47 nm to 136.8 ± 2.9 nm (*p* ≤ 0.01). By 4 h, it further increased sharply to 197.36 ± 5.19 nm, representing an increase of approximately 80 nm compared to the initial value (*p* ≤ 0.0001) (Figure 2B). Meanwhile, the drug concentration changes in the CUR-NLCs group were consistent with those of CUR in gastric juice, but the drug content decreased significantly by approximately 5% (*p* ≤ 0.0001, Figure 2E), although the pH remained stable (ΔpH < 0.03, Figure 2D).

In terms of storage stability, when CUR-NLCs were stored at 25 °C and 4 °C for 15 days and measured on days 0, 7, and 15, no significant changes were observed in average particle size or PDI (*p* > 0.05), demonstrating good storage stability of the formulation under the investigated conditions (Figure 2C).

The in vitro release profile (Figure 2F) showed that the release behavior of the CUR-NLCs group was slower than that of the CUR-Sol group. While the CUR-Sol group released over 90% of the drug within 24 h, the CUR-NLCs group released less than 55% during the same period, indicating that the NLCs significantly delayed the release of CUR (*p* ≤ 0.0001).

### 3.3. Lymphatic Transport and Pharmacokinetic Evaluation of CUR-NLCs

**Figure 3:**
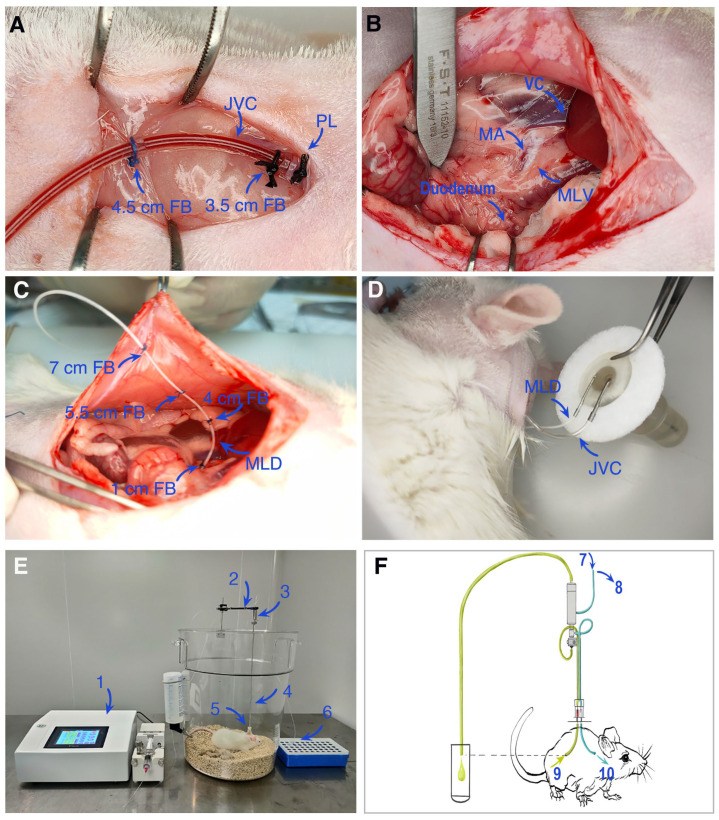
Schematic diagram and experimental setup of the mesenteric lymph duct and jugular vein catheterization model in rats. (**A**) Cannulation of the jugular vein for fluid administration or blood collection. (**B**,**C**) Cannulation of the mesenteric lymph duct for lymph collection. (**D**,**E**) Fluid collection devices for lymph and blood, respectively. (**F**) Schematic overview of the entire experimental apparatus. Abbreviations: FB, fixation buckle; JVC, jugular vein catheter; PL, proximal ligation; VC, vena cava; MA, mesenteric artery; MLV, mesenteric lymph vessel; MLD, mesenteric lymph duct; JV, jugular vein; 1, micro-infusion pump; 2, counterbalance arm; 3, 2-channel swivel; 4, VAB tether; 5, VAB; 6, lymph fluid collection tube; 7, reservoir of Ringer’s solution; 8, jugular vein blood collection tube; 9, mesenteric lymph vessel; 10, jugular vein; Blue tube: jugular vein catheter; Green tube: Mesenteric lymphatic duct.

This study established a rat mesenteric lymph duct-jugular vein assisted reflux model (Figure 3) in which cannulations were performed in the jugular vein (Figure 3A) and the mesenteric lymph duct (Figure 3B,C), connected to a fluid supplementation and collection device (Figure 3D,E). A complete schematic diagram of the experimental setup is shown in Figure 3F. Using this system, blood and lymph samples were collected and analyzed by LC-MS/MS to systematically compare the pharmacokinetic behavior after oral administration of CUR-Sol and CUR-NLCs.

**Figure 4:**
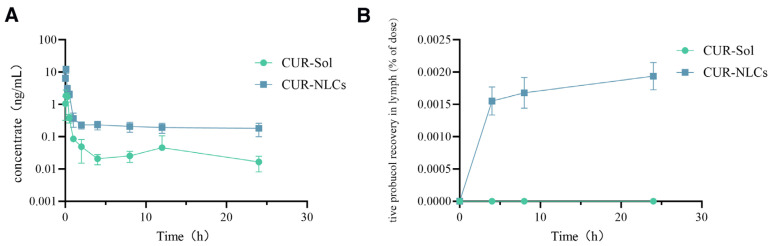
(**A**): Pharmacokinetic data from rat jugular vein; (**B**): Rat lymphatic fluid data.

The plasma concentration-time curve is shown in Figure 4A. Relevant data were processed using WinNonlin 6.2 software with a non-compartmental model, and the pharmacokinetic parameters are listed in Table 2. The results showed that, although the time to peak concentration (T_max_) was 5 min in both the CUR-Sol and CUR-NLCs groups, the CUR-NLCs group exhibited a significantly enhanced absorption effect. The peak concentration (C_max_) increased from 2.33 ± 0.5 ng/mL in the CUR-Sol group to 12.23 ± 3.07 ng/mL in the CUR-NLCs group, an approximately 5.25-fold improvement (*p* < 0.001). Additionally, CUR-NLCs significantly prolonged the drug retention time in vivo, with the elimination half-life (T_1_/_2_) extended from 15.0 ± 2.94 h in the CUR-Sol group to 33.49 ± 6.9 h in the CUR-NLCs group, an approximately 2.23-fold increase (*p* < 0.001). In terms of relative bioavailability, the area under the curve AUC_0_-t (ng·h/mL) of the CUR-NLCs group was significantly higher than that of the CUR-Sol group, with bioavailability improved approximately 5.13-fold (*p* < 0.001), further confirming a significant enhancement in oral absorption efficiency.

This study employed a mesenteric lymph duct-jugular vein auxiliary reflux model to directly evaluate the lymphatic transport characteristics of the drug. As shown in Figure 4B and Table 3, drug distribution was detectable in the lymph of the CUR-NLCs group, while no drug was detected in the CUR-Sol group throughout the sampling period. The results indicate that CUR-NLCs can be absorbed via the intestinal lymphatic pathway, thereby bypassing hepatic first-pass metabolism and improving oral bioavailability.

To investigate whether CUR-NLCs are absorbed via the intestinal lymphatic pathway, 18 SPF-grade female ICR mice were randomly divided into three groups and orally administered normal saline (NA group), CUR-Sol, or CUR-NLCs (at a dose of 5 mg/kg), respectively. 5 min after administration, jejunal tissues were collected and processed through fixation, dehydration, and ultrathin sectioning for observation under transmission electron microscopy.

**Figure 5:**
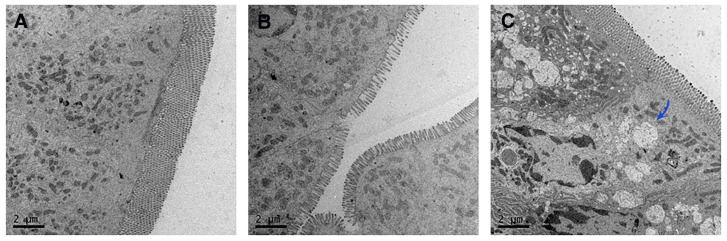
(**A**): Chylomicrons observed by TEM in the normal saline group; (**B**): Chylomicrons observed by TEM in the CUR-Sol group; (**C**): Chylomicrons observed by TEM in the CUR-NLCs group. Note: The blue arrow points toward chylomicrons.

The results revealed significant differences in lipid transport characteristics among the jejunal tissues of each group. No obvious chylomicron formation was observed in the intestinal epithelial cells of either the normal saline control group (NA group, Figure 5A) or the free CUR-Sol group (Figure 5B). In contrast, abundant typical chylomicron aggregates, ranging from 0.1–2 μm in size (as indicated by black arrows), were observed in the cytoplasm and at the basolateral membrane of intestinal epithelial cells in the CUR-NLCs group (Figure 5C).

## 4. Discussion

NLCs, as second-generation lipid-based nanocarriers, offer high drug loading, physical stability, and biocompatibility and have shown promise in enhancing the oral absorption of poorly soluble drugs such as curcumin. However, the exact absorption mechanism, particularly the role of intestinal lymphatic transport, remains unclear, as most studies rely on indirect evidence that cannot distinguish systemic lymphatic uptake from tissue retention. We developed a simple, efficient lymph-targeting CUR-NLCs formulation and confirmed its lymphatic transport in conscious rats using a mesenteric lymph duct-jugular vein shunt model. The results confirmed that CUR-NLCs bypass hepatic first-pass metabolism, significantly enhancing systemic exposure.

The superior performance of CUR-NLCs can be attributed to three key physicochemical properties. First, the spherical particle size of approximately 100 nm promotes intestinal absorption. Particles smaller than 200 nm, due to their larger surface area and appropriate size, adhere more effectively to the intestinal epithelium and enhance cellular uptake, facilitating efficient drug absorption [25]. Incorporation of CUR led to a slight but consistent increase in particle size compared with blank NLCs, likely due to encapsulation and interfacial anchoring of CUR molecules within the lipid matrix, which causes minor expansion of the nanoparticle structure [26]. Second, CUR-NLCs exhibit an exceptionally high encapsulation efficiency of 99.99%, outperforming most reported CUR delivery systems. For example, whey protein-stabilized emulsions achieved 96.26% under optimal conditions [27], and a casein-dextran sulfate nanocomplex reached 94.8% through a multi-step pH-adjusted process [28]. In contrast, our simpler method maintains outstanding packaging efficiency while effectively preventing drug leakage. Finally, the negatively charged surface (Zeta potential −14.14 mV) contributes to stability and efficacy. This charge primarily originates from the anionic phospholipid groups [29]. The increased absolute Zeta potential of CUR-NLCs compared with blank NLCs likely results from CUR molecules anchoring at the lipid interface via hydrogen bonding with phospholipid head groups [30]. This enhances surface negativity, providing two main advantages: (1) improved colloidal stability by increasing electrostatic repulsion and preventing aggregation, in accordance with DLVO theory [31]; (2) optimized intestinal retention, as moderate negative charge facilitates electrostatic interactions with positively charged microdomains in the mucus while avoiding excessive clearance, thereby prolonging residence time and enhancing drug absorption [32].

In the simulated gastrointestinal stability study, CUR-NLCs exhibited excellent physicochemical stability in gastric conditions. After 4 h incubation in SGF, particle size varied by less than 8 nm, and although minor fluctuations in drug content were observed, no significant overall change occurred, indicating that the lipid nanoparticles maintained structural integrity under acidic and enzymatic conditions. Upon transition to the intestinal environment, CUR-NLCs displayed more dynamic behavior. In SIF, an initial burst release was observed within the first 0.5 h, likely due to rapid diffusion of drug molecules adsorbed on the nanoparticle surface. Between 0.5 and 1 h, a transient increase in CUR concentration occurred, possibly reflecting dynamic reorganization of the lipid matrix, leading to partial re-adsorption or structural compaction affecting measured analyte levels [33]. From 1 to 4 h, particle size increased significantly, accompanied by gradual drug leakage, suggesting that intestinal enzymes and bile salts progressively disrupted the nanoparticle structure, consistent with previous observations of nanoemulsion behavior in the gut [34,35]. Despite these dynamic changes, CUR-NLCs achieved sustained release, with cumulative drug release remaining below 55% over 24 h. This behavior aligns with other CUR nano-carriers, such as IRMOF-8 [36] and rhamnolipid-modified liposomes [37], demonstrating the ability of appropriate nanocarrier strategies to maintain stable intestinal drug concentrations. Such sustained release is crucial for prolonging therapeutic effect and reducing dosing frequency.

The pharmacokinetic analysis revealed that CUR-NLCs markedly improved the oral absorption and systemic persistence of curcumin. Compared with the CUR-Sol group, CUR-NLCs increased the peak plasma concentration (C_max_) by 5.25-fold and the area under the concentration-time curve (AUC_0–t_) by 5.13-fold, indicating substantially enhanced drug absorption and systemic exposure. The elimination half-life (T_1/2_) was prolonged to 33.49 h, approximately 2.23 times that of CUR-Sol, suggesting that the nano-carrier delayed drug clearance via sustained release and altered tissue distribution. A key finding of this study was the direct demonstration of intestinal lymphatic transport. Using a mesenteric lymph duct–jugular vein shunt model in conscious rats, CUR was consistently detected in the lymph of the CUR-NLCs group, whereas no drug was observed in the CUR-Sol group. This pharmacokinetic evidence was further corroborated by TEM observations: abundant chylomicrons (0.1–2 μm) were present within the jejunal epithelium of mice treated with CUR-NLCs but absent in the control group. Given that chylomicrons are natural carriers for lipids and lipophilic compounds, their presence confirms that CUR-NLCs successfully engage the intestinal lymphatic pathway [38]. Together, the pharmacokinetic and morphological data provide complementary evidence that CUR-NLCs exploit the lymphatic system to bypass hepatic first-pass metabolism, directly entering systemic circulation and substantially enhancing oral bioavailability. These findings offer critical insights for the rational design of more efficient oral nano-drug delivery systems.

Although this study successfully demonstrated the lymph-targeting and oral bioavailability-enhancing effects of CUR-NLCs, certain limitations remain. First, the investigation was limited to a short-term stability assessment of 15 days, which ensured the consistency and initial integrity of the formulation during the experimental period. However, its long-term stability, particularly the physicochemical changes over clinically relevant periods such as 6 or 12 months, remains unclear and warrants further investigation. Second, the current formulation exhibits a relatively low drug loading capacity. Although the high encapsulation efficiency ensures effective drug retention, the low drug loading requires the administration of a larger number of excipients to achieve a therapeutically relevant dose of CUR. This may increase the potential risk of excipient-related toxicity and could limit its application in high-dose therapeutic scenarios.

## 5. Conclusions

In summary, this study provides a practical strategy for developing lymphatic-targeted delivery systems for hydrophobic drugs with low oral bioavailability. It also offers mechanistic insights into lymphatic absorption and highlights NLCs as a promising platform for oral delivery of such compounds. Future work will focus on optimizing the formulation to enhance drug loading and long-term storage stability, evaluating therapeutic efficacy in relevant disease models, characterizing curcumin metabolites to better understand in vivo metabolism, and extending the platform to other poorly soluble drugs to explore its broader applicability.

## Figures and Tables

**Table 1:** Physicochemical characteristics of NLCs.

Formulation	Size (nm)	PDI	Zeta (mV)	DL (%)	EE (%)
Blank-NLCs	95.94 ± 1.22	0.20 ± 0.01	−7.93 ± 0.33		
CUR-NLCs	117.28 ± 1.32	0.20 ± 0.02	−14.14 ± 0.30	1.73	99.99

**Table 2:** Pharmacokinetic parameters of the whole blood of rats in each group.

Parameters	CUR-Sol	CUR-NLCs
C_max_ (ng/mL)	2.33 ± 0.50	12.23 ± 3.07 ****
T_max_ (h)	0.083	0.083
T_1/2_ (h)	15.0 ± 2.94	33.49 ± 6.90 ***
AUC_0–t_ (ng·h/mL)	1.52 ± 0.59	7.81 ± 2.20 ****
AUC_0–∞_ (ng·h/mL)	1.83 ± 0.70	14.5 ± 5.20 ****

Note: *** *p* < 0.001 and **** *p* < 0.0001.

**Table 3:** Dose percentage parameters of lymphatic fluid of rats in all group.

Parameters	Liquid Collection Time, h	CUR-Sol	CUR-NLCs
Concentrate, ng·mL^−1^	0–4	0	5.05 ± 0.24
	4–8	0	0.41 ± 0.03
	8–24	0	0.23 ± 0.04
V, mL	0–4	4.78 ± 0.54	4.64 ± 0.33
	4–8	4.99 ± 0.48	4.48 ± 0.71
	8–24	16.71 ± 0.86	16.60 ± 0.66
Dose percentage, %	0–4	0	0.0013 ± 0.0001
	4–8	0	0.0014 ± 0.0001
	8–24	0	0.0016 ± 0.0001

## Data Availability

The original contributions presented in this study are included in the article. Further inquiries can be directed to the corresponding author.

## Abbreviations

The following abbreviations are used in this manuscript:

CUR	Curcumin
CUR-NLCs	Curcumin-loaded nanostructured lipid carrier
CUR-Sol	Curcumin Solution
LC-MS/MS	Liquid Chromatography-Tandem Mass Spectrometry
SPF	Specific Pathogen Free
SD	Sprague Dawley
DLS	dynamic light scattering
EE	Encapsulation Efficiency
DL	Drug Loading
SGF	Simulated Gastric Fluid
SIF	Simulated Intestinal Fluid
VAB	Vascular Access Button
FB	fixation buckle
JVC	jugular vein catheter
PL	proximal ligation
VC	vena cava
MA	mesenteric artery
JV	jugular vein
CO_2_	carbon dioxide
